# Extreme drought pushes stream invertebrate communities over functional thresholds

**DOI:** 10.1111/gcb.14495

**Published:** 2018-11-14

**Authors:** Thomas W. H. Aspin, Kieran Khamis, Thomas J. Matthews, Alexander M. Milner, Matthew J. O’Callaghan, Mark Trimmer, Guy Woodward, Mark E. Ledger

**Affiliations:** ^1^ School of Geography, Earth and Environmental Sciences University of Birmingham Birmingham UK; ^2^ Wessex Water Bath UK; ^3^ 2CE3C – Centre for Ecology, Evolution and Environmental Changes/Azorean Biodiversity Group, Depto de Ciências Agráriase Engenharia do Ambiente Universidade dos Açores, Angra do Heroísmo Açores Portugal; ^4^ Institute of Arctic Biology University of Alaska Fairbanks Alaska; ^5^ School of Biological and Chemical Science Queen Mary University of London London UK; ^6^ Department of Life Sciences Imperial College London Ascot Berkshire UK

**Keywords:** climate change, disturbance gradient, drought, ecological threshold, functional traits, macroinvertebrates, stream drying

## Abstract

Functional traits are increasingly being used to predict extinction risks and range shifts under long‐term climate change scenarios, but have rarely been used to study vulnerability to extreme climatic events, such as supraseasonal droughts. In streams, drought intensification can cross thresholds of habitat loss, where marginal changes in environmental conditions trigger disproportionate biotic responses. However, these thresholds have been studied only from a structural perspective, and the existence of functional nonlinearity remains unknown. We explored trends in invertebrate community functional traits along a gradient of drought intensity, simulated over 18 months, using mesocosms analogous to lowland headwater streams. We modelled the responses of 16 traits based on a priori predictions of trait filtering by drought, and also examined the responses of trait profile groups (TPGs) identified via hierarchical cluster analysis. As responses to drought intensification were both linear and nonlinear, generalized additive models (GAMs) were chosen to model response curves, with the slopes of fitted splines used to detect functional thresholds during drought. Drought triggered significant responses in 12 (75%) of the a priori‐selected traits. Behavioural traits describing movement (dispersal, locomotion) and diet were sensitive to moderate‐intensity drought, as channels fragmented into isolated pools. By comparison, morphological and physiological traits showed little response until surface water was lost, at which point we observed sudden shifts in body size, respiration mode and thermal tolerance. Responses varied widely among TPGs, ranging from population collapses of non‐aerial dispersers as channels fragmented to irruptions of small, eurythermic dietary generalists upon extreme dewatering. Our study demonstrates for the first time that relatively small changes in drought intensity can trigger disproportionately large functional shifts in stream communities, suggesting that traits‐based approaches could be particularly useful for diagnosing catastrophic ecological responses to global change.

## Graphical abstract

A growing number of studies are using species' traits to diagnose and predict their responses to long‐term climate change, but few have explored trait sensitivity to extreme climatic events, such as prolonged droughts. We combined a large manipulative flow experiment with traits‐based analyses to elucidate functional responses of stream macroinvertebrate communities to intensifying drought. Drought caused abrupt and pervasive changes in community trait profiles and triggered population collapses of certain functional groups, most notably those comprising non‐flying taxa. Our detection of multiple response thresholds indicates that traits could be valuable functional biomarkers for species‐ and community‐level resistance to extreme disturbance.

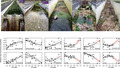

## 1 INTRODUCTION

Vulnerability assessments are increasingly using species’ functional traits to explain and infer their sensitivities to long‐term climate change (e.g., Domisch et al., 2013; MacLean & Beissinger, 2017; Pacifici et al., 2017; Pearson et al., 2014). Traits have less commonly been used to diagnose ecological responses to climatic extremes, which are projected to become more frequent and intense globally (Dai, 2013; Fischer & Knutti, 2015) and are less likely to offer opportunity for species adaptation (Poff et al., 2018; Thompson, Beardall, Beringer, Grace, & Sardina, 2013; Vázquez, Gianoli, Morris, & Bozinovic, 2017). Extreme events such as drought can push ecological communities beyond critical thresholds (Bailey & van de Pol, 2016), defined here as the point(s) along an environmental gradient where a relatively small change in conditions provokes a disproportionately large biotic response (Capon et al., 2015; Groffman et al., 2006; Kelly et al., 2015). Anticipating the ecological impacts of drought hinges on understanding when and why these thresholds are crossed (Standish et al., 2014). However, gradient‐based studies that can detect causal relationships and nonlinearities in the relevant response variables are largely lacking (Kreyling, Jentsch, & Beier, 2014).

In running waters, abrupt ecological responses to drought may be expected as critical habitats are lost, such as when the drying of riffles fragments the channel into isolated pools, or when the streambed dries completely (Boulton, 2003; Chadd et al., 2017). However, this nonlinearity has predominantly been explored with structural metrics (species richness, community composition), and it remains unclear whether thresholds can also be detected in the functional trait profiles of stream biota. By explicitly linking environmental perturbation to species response, functional traits can provide greater mechanistic understanding of disturbance impacts than taxonomic approaches (Chessman, 2015; Floury, Usseglio‐Polatera, Delattre, & Souchon, 2017), and as environment‐trait relationships potentially transcend biogeographic boundaries, they should yield more universally relevant findings (Menezes, Baird, & Soares, 2010; Schriever & Lytle, 2016; Walters, 2011). Moreover, traits‐based indices, particularly frequency distributions of individual traits, appear to be stronger indicators of ecosystem functioning than taxonomic composition (Gagic et al., 2015). A traits‐based approach to threshold detection therefore has the potential to significantly improve our understanding of drought, providing (a) information on the key biological mechanisms driving abrupt community shifts; (b) transferable observations of species’ vulnerabilities to critical habitat loss; and (c) insights into when and how community functioning may be most affected (Dézerald, Céréghino, Corbara, Dejean, & Leroy, 2015).

Traits‐based studies in freshwaters have primarily focused on macroinvertebrates, reflecting their wide distribution, high diversity and prominent role in ecosystem functioning (Menezes et al., 2010). Various studies have explored macroinvertebrate trait responses to hydrologic disturbance (e.g., Bêche, Mcelravy, & Resh, 2006; Bonada, Dolédec, & Statzner, 2007; Schriever et al., 2015; Leigh et al., 2016), but these have overwhelmingly investigated seasonal drying events which do not represent true extremes for their locale, and to which species are preadapted with a suite of suitable traits and coping mechanisms (Lytle & Poff, 2004). For instance, in environments with a history of severe drying, the strongest biological changes are typically delayed until surface water is completely lost, reflecting local biotic adaptation to all but the most severe disturbance (Boersma, Bogan, Henrichs, & Lytle, 2014; Bogan, Hwan, Ponce, & Carlson, 2017). Community resistance to extreme drought is typically much lower (Lake, 2003), and such events could therefore trigger marked ecological responses long before the streambed dries. We might expect the timing of any such responses to be trait‐specific, with changes in species behaviour as drought initially intensifies giving way to subsequent shifts in morphology and physiology, as survival becomes progressively more difficult without physiological adaptations to drying (Hershkovitz & Gasith, 2013; Stubbington & Datry, 2013).

Despite observed and projected increases in the frequency of extreme droughts, such events are still rare in running waters, creating an urgent need for large‐scale experiments which can expose species to novel conditions beyond their evolutionary envelopes (Kayler et al., 2015; Knapp et al., 2017; Ledger & Milner, 2015). Furthermore, most definitions of an ecological threshold relate the rate of change in ecosystem state to that of a specific environmental pressure in isolation (Capon et al., 2015; Groffman et al., 2006). This is difficult or impossible to validate as a causal driver‐response relationship in correlational studies, which are often beset by confounding influences beyond the stressor of interest, and instead favours detection in an experimental setting (Kayler et al., 2015; Kreyling et al., 2014). Mesocosms are thus suitable as they can isolate trait responses to stream drought from possible confounding factors (Woodward et al., 2016), such as changing pollutant levels, underlying climatic and hydrological regimes and other site‐specific contingencies, including surrounding land use (Ding et al., 2017; Durance & Ormerod, 2009; Floury et al., 2017; Thomson et al., 2012; Yao et al., 2017). Crucially, of all experimental approaches, mesocosms also allow for the greatest compromise between realism and replicability (Stewart et al., 2013).

We therefore tested for thresholds in the responses of macroinvertebrate traits across an experimental gradient of drought intensification that encompassed several critical stages of habitat loss. Here, we use the term threshold in a statistical sense, namely a stage in a relationship where the response variable changes more rapidly than the predictor (Groffman et al., 2006; Kelly et al., 2015; Yin, Leroux, & He, 2017). Statistically robust ecological threshold detection methods are commonly used to gauge maximum permissible levels of habitat fragmentation in terrestrial ecosystems (Swift & Hannon, 2010), but have received relatively little attention in the aquatic realm (King & Baker, 2014). Such detection methods nonetheless offer a potentially powerful tool for freshwater ecologists since, by fragmenting habitat, stream drying broadly mimics the impacts of land‐use disturbances. Recognizing that individual traits typically covary, as a product of trait coevolution and fitness trade‐offs (Menezes et al., 2010; Poff et al., 2006), we used two separate approaches. We firstly analysed 16 individual traits with clear, established linkages to drought, thus minimizing the possibility of observing spurious environment‐trait relationships (Pilière et al., 2016; Verberk, Noordwijk, & Hildrew, 2013). We then explicitly accounted for trait intercorrelations by grouping taxa according to their trait profiles and analysing responses of these trait profile groups (TPGs) to drought (following Pilière et al., 2016). Our study thus comprised both readily interpretable observations of community‐weighted individual traits and models of complete trait profiles.

For all individual traits analysed, we made a priori predictions of functional responses to drought (see Table 1), which were ancillary to three overarching hypotheses. These were formulated on the basis that trait selection is likely to shift abruptly as drought intensifies and habitats are lost, and were as follows: (1) moderate‐intensity droughts (pool habitat fragmentation) would predominantly trigger responses in behavioural traits (e.g., dispersal, locomotion); whereas (2) under high drought intensity (streambed drying), changes in morphology and physiology (e.g., towards dessication resistant forms and aerial respiration) would also be apparent; and (3) individual trait and/or TPG responses to drought would be highly nonlinear, with some thresholds detected before complete surface water loss.

**Table 1:** Expected impacts of drought on the 16 a priori‐selected traits

Grouping feature	Trait (response to drought)	Rationale	Reference(s)
Body size	Small: <0.1 mg (↑)	Drought favours small taxa with low metabolic demands and easy access to refugia relative to intermediate and large body sizes	Griswold et al. (2008), Ledger et al. (2011), Woodward et al. (2016)
	Medium: 0.1–1 mg (↓)		
	Large: 1–2 mg (↓)		
	Vlarge: >2 mg (↓)		
Voltinism	Multivoltine (↑)	High reproductive rate maximizes chance of recruitment success	Díaz, Alonso, and Gutiérrez (2008), Chessman (2015), Schriever and Lytle (2016)
Reproduction	Ovoviviparous (↑)	Ovoviviparity reduces risk of egg mortality in stressful conditions	Díaz et al. (2008), Floury et al. (2017)
Resistance	Resistant (↑)	Resistance forms reduce vulnerability to dessication	Bêche et al. (2006), Bonada, Dolédec, et al. (2007), Griswold et al. (2008), Robson, Chester, and Austin (2011)
Dispersal	Active aerial (↑)	Active aerial dispersal enables regular recolonization of disturbed habitats; recolonization by active aquatic dispersers is limited as channels fragment	Bonada, Dolédec, et al. (2007), García‐Roger et al. (2013), Cid et al. (2016), Schriever and Lytle (2016)
	Active aquatic (↓)		
Locomotion	Crawling (↓)	Crawlers are vulnerable to predation in shrinking pools and dessication upon water loss; burrowers are better able to access streambed refugia and survive fine sediment deposition	Bonada, Rieradevall, and Prat (2007), Díaz et al. (2008), Griswold et al. (2008), Robson et al. (2011), Walters (2011), Vadher, Leigh, Millett, Stubbington, and Wood (2017)
	Burrowing (↑)		
Respiration	Tegument (↓)	Oxygen depletion in shrinking pools and loss of water favour aerial over tegument respiration	Bonada, Dolédec, et al. (2007), Bonada, Rieradevall, et al. (2007), Robson et al. (2011)
	Spiracle (↑)		
Diet	Generalist (↑)	Taxa with broad dietary preferences are better adapted to cope with prey loss/resource shortages during drought	Williams (1996), Vázquez and Simberloff (2002)
Thermal preference	Cold: <15°C (↓)	Eurythermic taxa are more tolerant of water temperature extremes during drought	Chessman (2015, 2018)
	Eurythermic (↑)		

Note

Body size classes were assigned based on body mass estimates (mg dry mass).

## 2 MATERIALS AND METHODS

### 2.1 Study site and experimental design

The research was undertaken over 2 years (February 2013–January 2015) across 21 stainless steel, flow‐through stream mesocosms (spring‐fed headwater stream analogues, each 15 m × 0.5 m × 0.5 m). These were sited next to a perennial reach of the Candover Brook, a mesotrophic chalk stream in the River Itchen catchment, Hampshire, UK (51°10′21″N, 1°18′70″W). Initially, borehole water was pumped into each mesocosm (to capacity) through an inlet pipe and drained over an outlet weir. Our outdoor, once‐through setup thus followed design recommendations for maximizing the physicochemical and biological realism of stream mesocosms (Ledger, Harris, Armitage, & Milner, 2009). Bed material comprised fine and coarse gravel distributed to create alternating sections of deep and shallow habitat typical of lowland, low‐energy chalk streams (Sear, Armitage, & Dawson, 1999; Sear, Newson, & Thorne, 2004). In each mesocosm, we created three shallow sections using bed layer depths of 25 cm, and four deep sections using bed layer depths of 15 cm. This necessarily simplified design could not capture the full morphological and hydraulic complexity of natural riffle‐pool sequences, but it did include a core subset of properties that influence ecosystem responses to drought in field settings (i.e., variability of depth and substrate and associated refugia), thus allowing us to test for ecological responses to the progressive loss of critical stream habitat. Throughout the manuscript, we use the terms “riffle” and “pool” to denote shallow and deep sections of stream habitat, respectively, to ensure that our terminology is consistent with other studies (e.g., Boulton, 2003). Macrophytes (*Ranunculus penicillatus *subsp. *pseudofluitans* (Syme) S.D. Webster), algae and macroinvertebrates were collected from nearby perennial stream reaches to seed the channels with taxa from the regional species pool. The mesocosms were then left to run undisturbed for 6 months to allow for community development. The channels were also accessible to aerial colonists throughout the experiment, during both this pre‐disturbance period and the drought phase.

In August 2013, the sluices on the inlet pipes were adjusted to simulate a gradient of drought intensity, with each sluice maintained at a fixed setting throughout the remainder of the experiment (until January 2015) to sustain the gradient. Each channel represented a distinct treatment with a unique wetted area (range 6.5–0.25 m^2^), water volume (1.9–0.001 m^3^), flow (2.2–0.001 L/s) and temperature range (6–40°C maximum temperature range; Supporting Information Figure S1). During stream drought, these primary stressors covary to elicit physicochemical (e.g., oxygen availability, conductivity) and biological responses (Lake, 2011). The wide range of conditions we simulated was designed to expose the biota to levels of environmental stress beyond their typical limits, as recommended by Kayler et al. (2015) to infer potential responses to future climate extremes. Our gradient approach offered several advantages over a more conventional factorial design with true replicates, as it allowed us to rigorously test for thresholds (Kreyling et al., 2014) and conduct analyses with significantly greater statistical power (i.e., regression‐based vs. analysis of variance‐based; Cottingham, Lennon, & Brown, 2005).

Although groundwater‐fed chalk stream reaches are typically hydrologically stable (Sear et al., 1999), protracted dry weather can trigger extreme low flows, such as during the severe droughts of 1989–1992 and 2010–2012, when falling groundwater levels gave rise to prolonged periods of stagnation and streambed drying (Folland et al., 2015; Kendon, Marsh, & Parry, 2013; Westwood, Teeuw, Wade, & Holmes, 2006). Our supraseasonal drought experiment was designed to reproduce these extreme but realistic conditions, which are predicted to become more frequent given projected declines in groundwater recharge and baseflows under climate change (Jackson, Meister, & Prudhomme, 2011). Furthermore, the timing of our drought phase, beginning in summer and ending in winter, was realistic: in a groundwater‐dominated stream such an event could be triggered by rainfall deficits over two consecutive winters (Wood & Petts, 1999). Drought termination might plausibly then occur the following winter in response to increased autumn rainfall, reflecting the long hydrological lag times characteristic of chalk systems (Parry, Wilby, Prudhomme, & Wood, 2016).

### 2.2 Sampling and processing

In January 2015, we used a Surber sampler (0.0225 m^2^, mesh size 300 µm) to collect four benthic macroinvertebrate samples per channel (one sample per pool), which were then preserved in 70% industrial methylated spirit. Each sample comprised the uppermost 3 cm of bed gravel spanning the entire surface area of the Surber frame, allowing us to directly compare flowing and non‐flowing channels. In the most drought‐affected treatments, samples consisted of both dry and wet gravels: surface water was largely absent, but in the upper layer of substrate (<3 cm depth) interstitial refugia persisted and supported macroinvertebrates. Samples were taken only from pools as our focus was to compare aquatic habitats across the drought gradient: the riffle sections of over half of the treatments consisted of exposed, dry gravels. Moreover, our simplified riffle and pool habitats did not differ markedly in either flow profile (broadly uniform) or substrate type (clean gravel), and thus supported similar faunal assemblages. In the laboratory, we used a microscope to separate macroinvertebrates from detritus and identify specimens to genus (except Oligochaeta, which were recorded as such). Taxa were counted and abundance data from each of the four technical replicate samples were pooled and converted to a measure of density (individuals per m^2^).

We recorded water temperature at 15‐min intervals using Tinytag loggers (Gemini Data Loggers Ltd, Chichester, UK) placed in the terminal pool of each channel. Since oxygen depletion can be a critical stressor during stream drought (Lake, 2011), we also recorded dissolved oxygen (DO) levels in each stream at 5‐min intervals over one 24‐hr period each month using MiniDOT loggers (PME Inc., Vista, CA, USA) suspended midway through the water column. Temperature data were used to calculate the maximum recorded water temperature range, and oxygen data the mean daily minimum DO level, as environmental extremes are typically a stronger predictor of species’ responses than means (Vasseur et al., 2014; Vázquez et al., 2017).

### 2.3 Data analysis

#### 2.3.1 Abiotic variables

We used the axis one scores of a centred, covariance principal component analysis (PCA, explained variance = 94%) to integrate measurements of the four primary drought stressors (wetted area, water volume, flow, maximum recorded temperature range) into a compound index of drought intensity (DI; Supporting Information Table S1). The index was rescaled to vary from 0 (no drought disturbance) to 1 (most severe drought). Low DI (<0.2) was characteristic of channels that remained longitudinally connected, with minimal loss of wetted benthic habitat, stable temperatures (annual range <7.5°C) and relatively high flow (0.7–2.3 L/s; Supporting Information Figure S2). Moderate DI (0.2–0.7) described fragmented channels with dry riffles and isolated pools (mean 48% loss of wetted area), more variable temperatures (annual range 5–29°C) and negligible flow (<0.4 L/s). High DI (>0.7) denoted severe streambed dewatering (>95% loss of wetted area) accompanied by extreme temperature instability (annual range >38°C). The drought index thereby included two critical stages of habitat loss: (a) riffle drying/pool fragmentation and (b) pool drying (Supporting Information Figure S2b; Boulton, 2003). Consistent with these trends, we observed a broadly linear decline in minimum DO levels across the gradient (Supporting Information Figure S2e).

#### 2.3.2 Traits

Trait values were assigned at the genus level, using fuzzy‐coded information from the European trait databases published by Serra, Cobo, Grac, and Feio (2016) for Chironomidae and Tachet, Bournaud, Richoux, and Usseglio‐Polatera (2010) for all other taxa. Where the taxonomic resolution of trait information exceeded our identification level (e.g., Oligochaeta), we used the average trait profile of genera belonging to that taxonomic group (following Bêche et al., 2006). We selected 16 traits, straddling nine grouping features (sensu Schmera, Podani, Heino, Erős, & Poff, 2015) to test our a priori predictions of trait filtering by drought (Table 1). As reported body sizes in trait databases may show limited concordance with the true size distribution of specimens (Orlofske & Baird, 2014), we formulated more accurate size classes based on body mass estimates from our samples. Specimen body lengths were measured to the nearest 0.1 mm using an eyepiece graticule (minimum 30 randomly selected individuals per genus per sample for abundant taxa) and converted to body mass (mg dry mass) using published length‐mass regression equations with a bias towards European studies (Supporting Information Table S2). Body mass data from all channels were then aggregated to obtain size‐frequency distributions for each genus.

To test our prediction that drought would increase the proportion of generalists in the community (see Table 1), dietary information was condensed into a single trait that expressed affinity to a generalist diet. This was calculated as the number out of seven food types (microorganisms, fine organic matter, coarse organic matter, algae, plants, dead invertebrates and live invertebrates) consumed by each genus (following Chessman, 2015). Similarly, resistance was coded as a single trait, calculated as the number out of three major resistance strategies (resistant eggs/statoblasts, cocoons/housings and diapause) displayed.

Prior to trait selection, we normalized trait values so that they summed to 1 within each grouping feature, thus ensuring that each grouping feature was equally weighted. For analysis of individual trait responses, the trait‐by‐genus matrix was multiplied by ln(*n* + 1)‐transformed abundance data, thus obtaining the abundance‐weighted mean trait profile for the community of each channel. The trait values within each grouping feature were then again standardized to 0–1 so that they described relative trait occurrences (White, Hannah, Martin, Wood, & Beatson, 2017).

#### 2.3.3 Trait profile groups

To delineate TPGs, we used the same nine grouping features, but this time incorporated a greater number of traits (*n* = 30 vs. 16; Table 2) to group taxa based on comprehensive trait profiles, thus ensuring that the core traits of each genus were represented within each grouping feature. We applied Gower's distance‐based hierarchical cluster analysis (Pavoine, Vallet, Dufour, Gachet, & Hervé, 2009) using Ward's method to the normalized trait‐by‐genus matrix to identify clusters of taxa with similar trait profiles. Gower's distance was used in conjunction with Ward's method as a double‐centering of the Gower dissimilarity matrix indicated that the dissimilarities closely resembled Euclidean distances (after Bruno, Gutiérrez‐Cánovas, Sánchez‐Fernández, Velasco, & Nilsson, 2016). An iterative procedure was used to select the optimal number of clusters, distinguished by the highest analysis of similarities (ANOSIM) *R* value, which would indicate maximum dissimilarity among clusters (for parsimony, and to avoid overfitting, we set an upper limit of 10 clusters as a starting condition). Random forest analysis was used to identify the most important traits and grouping features in TPG selection. Importance was calculated using Gini impurity, which describes the impurity (i.e., classification contamination) produced by splitting a particular trait in two (e.g., high ovoviviparity vs. low ovoviviparity) at each node within a decision tree (Liaw & Wiener, 2015). We measured the importance of each trait for each TPG as the mean decrease in Gini impurity (hereafter Gini value), which computes the overall (forest‐wide) decrease in Gini impurity attributable to each trait (i.e., the higher the Gini value the more influential the trait).

**Table 2:** Traits upon which cluster analysis was performed to separate taxa into trait profile groups

Grouping feature	Trait
Body size	Small (<0.1 mg)
	Medium (0.1–1 mg)
	Large (1–2 mg)
	Vlarge (>2 mg)
Voltinism	Semivoltine
	Univoltine
	Multivoltine
Reproduction	Ovoviviparous
	Isolated eggs
	Clutches
	Asexual
Dispersal	Aquatic passive
	Aquatic active
	Aerial passive
	Aerial active
Resistance	Resistant
	Susceptible
Respiration	Tegument
	Gill
	Spiracle
Locomotion	Swimming
	Crawling
	Burrowing
	Interstitial
	Attached
Diet	Generalist
	Specialist
Thermal preference	Cold (<15°C)
	Warm (>15°C)
	Eurythermic

Note

The traits “susceptible” and “specialist” were calculated by subtracting the standardized “resistant” and “generalist” values from one.

#### 2.3.4 Statistical modelling

As trait responses to drought were highly nonlinear, we used generalized additive models (GAMs) to analyse the relationships between drought intensity and (a) trait occurrence (i.e., the standardized abundance‐weighted occurrence of a particular trait in the community) and (b) TPG abundance (i.e., the untransformed abundance of taxa belonging to a particular TPG, expressed as individuals per m^2^). Cross‐validation was used to guide the optimal level of smoothing (Wood, 2008) with minor modifications to avoid over‐smoothing, as recommended by Zuur, Ieno, Walker, Saveliev, and Smith (2009). GAMs were applied to rescaled data (see below), with diagnostic tests validating the choice of basis dimension for each smooth.

Where GAMs were significant (i.e., the *p*‐value of the smooth drought intensity term was lower than 0.05 following the Benjamini and Hochberg (1995) procedure for controlling the false discovery rate), thresholds were detected using the zonal habitat loss threshold approach of Yin et al. (2017). This method identifies thresholds as regions where the slope of the relationship between response and predictor (both rescaled to 0–1) is >1, thereby highlighting where a small change in environmental perturbation (here drought intensity) results in a larger change in community structure or function (here invertebrate traits). For clarity, we refer to this region as the threshold zone and to the critical lower bound of this zone, which marks the minimum level of disturbance required to induce a potentially catastrophic ecological response (Yin et al., 2017), as the breakpoint. The Yin et al. (2017) approach therefore provides a logical and elegant threshold detection method, using information on the slope of a relationship to identify thresholds in the strictest sense of the term (i.e., where the rate of change in a response variable exceeds that of a predictor; King and Baker (2014), Capon et al. (2015)). The method thus differs from detection approaches based on step functions, such as changepoint analysis, which more specifically test for regime shifts or alternative stable state transitions (King & Baker, 2014). Furthermore, GAMs are an effective tool for detecting ecological thresholds (Ficetola & Denoël, 2009), and by identifying a specific breakpoint (or breakpoints) the Yin et al. (2017) approach eliminates the subjectivity in threshold interpretation inherent in methods based on simple visual inspection of slopes (cf. Bino, Steinfeld, & Kingsford, 2014; Dézerald et al., 2015; White, McHugh, & McIntosh, 2016).

In accordance with the Yin et al. (2017) method, response data (trait occurrence and TPG abundance) were rescaled to vary from 0 to 1 before GAMs were fitted. We then used finite difference approximation (Eberly, 2016) to estimate the first derivative of the fitted spline of each GAM at 200 points along the drought gradient, and threshold zones were delineated where the first derivative was >1 or <−1. All analyses were undertaken in r (version 3.2.4) using the packages “ade4” (Dray, Dufour, & Thioulouse, 2017), “fd” (Laliberté, Legendre, & Shipley, 2015), “vegan” (Oksanen et al., 2017), “randomforest” (Liaw & Wiener, 2015) and “mgcv” (Wood, 2017).

## 3 RESULTS

### 3.1 Individual traits

**Figure 1:**
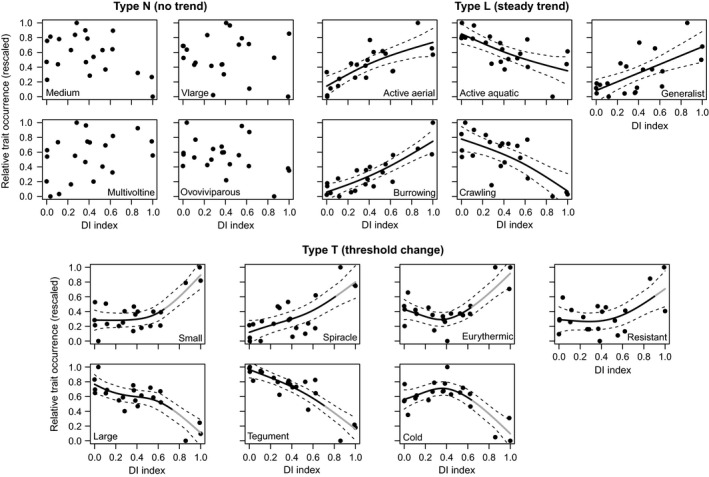
Relationships between drought intensity (DI) and relative occurrence of selected traits in the community (rescaled), grouped according to response type. Traits were selected from a priori predictions of responses to drought. Relationships are fitted with generalized additive models where significant (*p* < 0.05). Dashed lines are 95% confidence intervals. For Type T traits, grey sections of relationships denote threshold zones (slope >1 or <−1)

Of the 16 individual traits analysed, 12 (75%) responded significantly to drought intensification, with shifts towards smaller body sizes, aerial dispersal and respiration, burrowing habitat, generalist feeding, dessication resistance and broad thermal tolerance largely corroborating our a priori predictions of trait filtering (Table 3; Figure 1). Overall, three distinct response types were apparent across these individual traits. Four traits (medium body size, very large body size, multivoltinism and ovoviviparity) showed no significant response to drought (Type N response). Five traits (active aerial dispersal, active aquatic dispersal, burrowing, crawling and generalist diet) exhibited steadily increasing or decreasing (i.e., broadly linear) trends along the gradient (Type L response). Specifically, active aerial dispersal, burrowing and generalist feeding became gradually more prevalent as drought intensified, partly reflecting high proportions of taxa such as tanypod chironomids in fragmented channels and of other Diptera (primarily Ceratopogonidae) at high intensity. We observed corresponding, gradual decreases in active aquatic dispersal and crawling, largely driven by declining abundances of crustaceans, flatworms and leeches. These Type L response traits described species behaviour (dispersal, movement, feeding) which, in line with our first hypothesis, thus appeared to be sensitive to channel fragmentation as well as streambed drying.

**Table 3:** GAM output for significant relationships between drought intensity and both relative occurrence of individual traits and abundances of TPGs

Response variable	Response type (DI threshold)	*F*‐value	Deviance explained (%)
Individual traits			
Small	T (≥0.66)	11.9***	67.4
Large	T (≥0.74)	12.0***	68.2
Resistant	T (≥0.91)	4.38*	39.4
Active aerial	L	12.4***	58.8
Active aquatic	L	8.83**	48.7
Crawling	L	12.4***	53.9
Burrowing	L	26.8***	73.1
Tegument	T (≥0.71)	31.9***	79.7
Spiracle	T (≥0.82)	10.5***	57.9
Generalist	L	14.9***	43.9
Cold	T (≥0.64)	12.5***	68.6
Eurythermic	T (≥0.64)	12.2***	67.4
TPGs			
B	T (≤0.39)	28.0***	81.9
D	T (≥0.59)	21.2***	77.7
E	T (≤0.22)	10.4***	57.8
F	T (≥0.60)	52.0***	91.5
G	T (≤0.33)	11.6***	66.2

Note

Response type is linear (L) or threshold (T). The number in brackets after response type denotes the portion of the drought gradient where the slope of the fitted GAM is >1 or <−1. “Deviance explained” provides a measure of model performance, comparable to the *R* ^2^ value in ordinary regression. Significance value denotation is as follows: ns = non‐significant (*p* > 0.05); *p < 0.05; **p < 0.01; ***p < 0.001. All asterisked *F*‐values are significant (*p* < 0.05) following the Benjamini and Hochberg (1995) correction for controlling the false discovery rate. For complete results see Supporting Information Table S3.

The final seven traits (small body size, large body size, spiracle and tegument respiration, eurythermophily, cold‐adaptation and drought resistance) were characterized by thresholds in response (Type T response), with slight or no change under low‐moderate drought intensity but rapid change further along the gradient, with all breakpoints at DI values between 0.64 and 0.91. These breakpoints signalled shifts towards small body size (characteristic of most Diptera), spiracular respiration (typified by Psychodidae), wide temperature tolerance and high drought resistance (defining traits of e.g., Tipulidae and Ceratopogonidae). There was an abrupt and concomitant reduction in large body size and tegument respiration, partly reflecting declines in the most common caddisflies in the channels (*Drusus annulatus*, *Sericostoma personatum*) and in cold‐adaptation, which was particularly characteristic of *D. annulatus* and orthoclad chironomids. The responses of these traits thus corroborated our second main hypothesis, that shifts in morphology and physiology (e.g., size, respiration) would be most apparent upon streambed drying.

### 3.2 Trait profile groups

**Figure 2:**
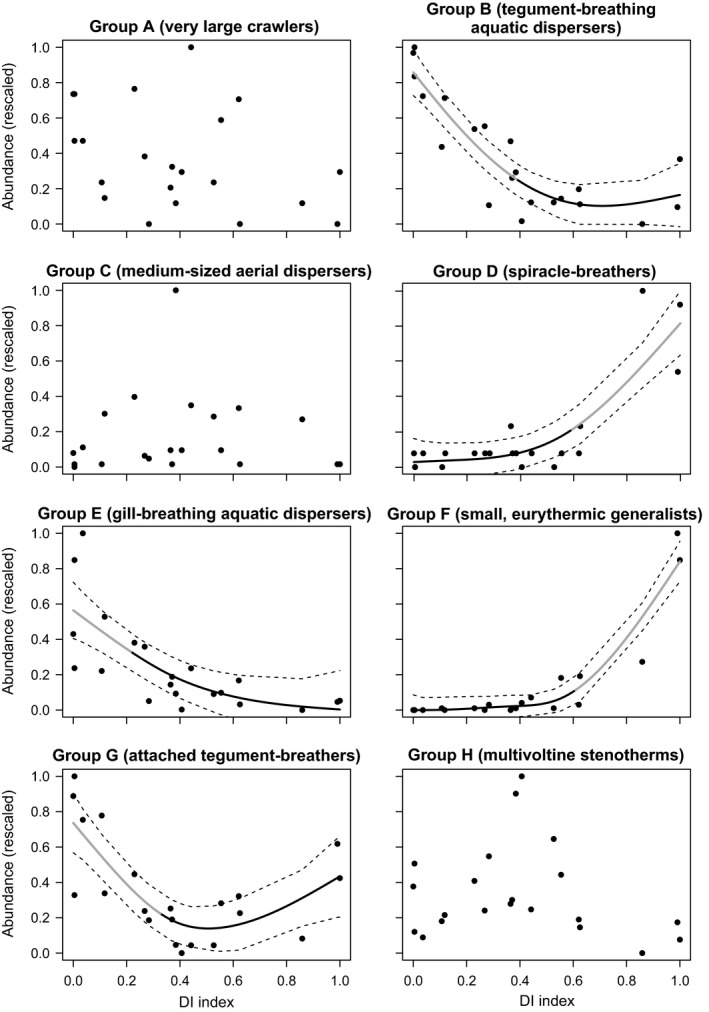
Relationships between drought intensity (DI) and rescaled abundances of trait profile groups (individuals per m^2^). Relationships are fitted with generalized additive models where significant (*p* < 0.05). Dashed lines are 95% confidence intervals. Grey sections of relationships denote threshold zones (slope >1 or <−1)

Cluster analysis identified eight TPGs (ANOSIM *R* value = 0.82; Table 4; Supporting Information Figure S3). The most important trait grouping features for partitioning genera into TPGs were thermal preference (Gini value = 2.46), body size (2.37) and respiration (2.29), followed by voltinism (2.12), diet (1.90), dispersal (1.53), locomotion (1.31) and reproduction (0.97; Supporting Information Figure S4). GAMs were significant for five TPGs, all of which exhibited thresholds in response to drought (Figure 2). Three groups (B, E and G), which contained aquatic dispersers and/or tegument‐breathers (primarily leeches/flatworms, crustaceans and worms/small caddisflies respectively; Table 4), were sensitive to low‐moderate intensity droughts and decreased rapidly in abundance across DI values ≤0.40. Two groups (D and F), which consisted of small, eurythermic aerial dispersers with either spiracle (e.g., Psychodidae) or gill (e.g., Ceratopogonidae) respiration, increased significantly in abundance under high‐intensity drought, with breakpoints at DI values of 0.59 and 0.60. The remaining TPGs (A, C and H), which comprised very large crawlers (e.g., large caddisflies, snails), medium‐sized aerial dispersers (e.g., Empididae) and multivoltine stenotherms (e.g., Orthocladiinae), respectively, displayed no significant trends along the gradient, though all were sensitive to high‐intensity drought. The responses of most TPGs were thus highly nonlinear, giving support to our third hypothesis, with the population collapses of groups B, E and G confirming our prediction that thresholds would not be confined to the high‐intensity part of the gradient.

**Table 4:** Overview of the eight TPGs identified by cluster analysis

TPG	Description	High affinity	Low affinity	Members
A	Very large crawlers	Vlarge (19.2)	Multivoltine (7.42)	*Drusus *(T)
		Generalist (5.38)		*Erpobdella *(H)
		Crawling (4.28)		*Potamophylax *(T)
		Aquatic active (4.05)		*Radix *(G)
				*Sericostoma *(T)
				*Sialis *(M)
				*Stagnicola *(G)
				*Tipula *(D)
B	Tegument‐breathing aquatic dispersers	Tegument (9.06)	Generalist (7.12)	*Dendrocoelum *(Tc)
		Aquatic active (8.06)	Multivoltine (6.71)	*Dugesia *(Tc)
		Crawling (7.28)		*Glossiphonia *(H)
				*Helobdella *(H)
				*Nemurella *(P)
				*Piscicola *(H)
				*Planaria *(Tc)
				*Polycelis *(Tc)
C	Medium‐sized aerial dispersers	Medium (6.06)		*Chelifera *(D)
		Aerial active (5.36)		*Clinocera *(D)
		Tegument (5.28)		*Elmis *(C)
		Clutches (4.71)		*Limnephilus *(T)
		Univoltine (3.62)		
D	Spiracle‐breathers	Spiracle (8.63)	Vlarge (3.12)	*Anopheles *(D)
		Clutches (1.93)	Attached (2.63)	*Metalimnobia *(D)
		Swimming (1.69)		*Pericoma *(D)
E	Gill‐breathing aquatic dispersers	Gill (4.20)	Aerial active (3.19)	*Asellus *(I)
		Multivoltine (3.40)	Clutches (3.19)	*Gammarus *(A)
		Crawling (2.85)		
F	Small, eurythermic generalists	Gill (8.72)		*Brachypogon *(D)
		Generalist (6.89)		*Culicoides *(D)
		Small (6.65)		*Palpomyia *(D)
		Multivoltine (6.22)		*Serratella *(E)
		Eurythermic (4.95)		*Serromyia *(D)
G	Attached tegument‐breathers	Attached (7.18)	Cold (7.27)	*Agapetus *(T)
		Tegument (5.93)	Vlarge (5.19)	*Chironomus *(D)
		Multivoltine (3.67)		*Oligochaeta*
				*Oxyethira *(T)
				*Plectrocnemia *(T)
				*Prodiamesa *(D)
				*Synorthocladius *(D)
H	Multivoltine stenotherms	Cold (16.7)	Vlarge (7.33)	*Brillia *(D)
		Multivoltine (9.30)		*Corynoneura *(D)
		Tegument (8.53)		*Cricotopus *(D)
		Aerial passive (8.16)		*Heterotrissocladius *(D)
				*Hydroptila *(T)
				*Krenopelopia *(D)
				*Limnophyes *(D)
				*Macropelopia *(D)
				*Metriocnemus *(D)
				*Micropsectra *(D)
				*Procladius *(D)

Note

The third and fourth columns list the five traits with which each group has the highest and lowest association, respectively. The numbers in brackets are measures of the decrease in Gini impurity resulting from taking the trait into account (the higher the number, the more influential the trait in delineating the TPG). The final column gives the genera belonging to each TPG, as well as the order to which the genus belongs (A = Amphipoda, C = Coleoptera, D = Diptera, E = Ephemeroptera, G = Gastropoda, H = Hirudinea, I = Isopoda, M = Megaloptera, P = Plecoptera, T = Trichoptera, Tc = Tricladida).

## 4 DISCUSSION

This study is the first to show that small differences in drought intensity can produce marked functional dissimilarities between stream communities, and that drought can prompt population crashes of certain functional groups with relatively limited (<50%) loss of wetted habitat. Many of the individual traits we analysed are closely tied to specific functions in stream ecosystems (e.g., aerial dispersal to resource subsidy provisioning; Ruhi, Dong, McDaniel, Batzer, & Sabo, 2018) or to fundamental network properties (e.g., dietary breadth to food web robustness; Nuwagaba, Zhang, & Hui, 2017). The significant trait responses reported here thus highlight the potentially pervasive impacts extreme droughts may have on stream community functioning. Furthermore, the responses of Type L traits and the population collapses of TPGs B, E and G suggest that these impacts are unlikely to be contingent on the disappearance of surface water per se, generally recognized as the most critical stage of habitat loss for stream biota (Boersma et al., 2014; Boulton, 2003).

Moderate‐ and high‐intensity droughts were associated with distinct changes in community trait profiles. Our findings suggest that drought‐driven habitat losses represent nested trait filters, with channel fragmentation and streambed drying both selecting for suitable behavioural traits but only the latter invoking high physiological resistance. It should be noted that these results could be conservative, as the communities of higher‐energy streams with greater numbers of specialist riffle‐dwellers (torrenticoles and rheophiles) might also display functional responses before the fragmentation stage (Boulton, 2003; Boulton & Lake, 2008). Here, some of the traits that became more prevalent as channels fragmented could have been a response to escalating biotic stress (e.g., burrowing as a predator avoidance mechanism, generalist feeding to cope with resource depletion), reflecting the potential for species’ interactions to intensify as wetted habitat shrinks (Boulton, 2003; Lake, 2003; McIntosh et al., 2017). The abrupt shifts in morphology/physiology at the more extreme end of the gradient are more likely to reflect environmental filters sensu* stricto* (Kraft et al., 2015). Such shifts are consistent with the results of a separate analysis, where functional turnover patterns indicated that severe dewatering gave rise to resistance strategies uncompetitive at lower levels of disturbance (Aspin et al., 2018). Few studies to date have analysed how trait selection evolves along a continuous stress gradient, hampering our ability to formulate general predictions regarding species’ sensitivities to intensifying extremes. Although continua of stressors are increasingly being described across natural streams (e.g., Ligeiro et al., 2013; Poff et al., 2018), the need to determine cause and effect in environment‐trait linkages (Poff et al., 2006) highlights the value of our mesocosm approach.

TPG responses to drought were largely consistent with theoretical predictions of life history strategies under varying degrees of disturbance (Verberk et al., 2013). The TPGs most sensitive to drought (B and E) comprised relatively large taxa with low dispersal ability, such as crustaceans and leeches, suggestive of life history strategies built around the dominance of stable resources (one of the core strategies outlined by Verberk et al. (2013)). By contrast, TPGs D and F, dominated by Diptera, were characterized by small body size, active aerial dispersal and generalist feeding, indicating life history strategies adapted to the exploitation of ephemeral resources in unpredictable, unstable environments (Verberk et al., 2013). With such strategies, taxa in these TPGs were successful colonizers of dry streambeds. The population collapses of TPGs B and E at relatively low drought intensity suggest that dispersal mode may be a critical determinant of the ability of a population to persist during severe drought, particularly in the face of a disturbance that exceeds generation time. Previous studies have similarly emphasized the important role of dispersal ability in mediating the effects of environmental variability on stream communities (Cañedo‐Argüelles et al., 2015; Lancaster & Downes, 2017; Patrick & Yuan, 2017; Schriever & Lytle, 2016), but few have demonstrated its impact in an experimental context free from potentially confounding drivers.

The ability to disperse to more favourable habitats may partly explain why the resistance of stream invertebrates to severe drying (ability to endure drought stress) is typically much lower than their resilience (resistance plus capacity to recover following flow resumption, sensu Hodgson, McDonald, & Hosken, 2015; Acuña et al., 2005; Boersma et al., 2014; Datry et al., 2014). However, recent studies of intermittent streams (Stubbington & Datry, 2013; Stubbington, Gunn, Little, Worrall, & Wood, 2016) have revealed viable life stages in dry bed sediments, indicating higher resistance than previously thought. The responses of TPGs D and F suggest that such resistance may extend to perennial stream communities. However, the success of these groups was not attributable solely to physiological resistance mechanisms: active aerial dispersal and burrowing habit were most prevalent in dewatered channels, indicating that regular recolonization from external lentic and semi‐aquatic source habitats adjacent to our mesocosms (e.g., ponds, drainage ditches, wet soils) and access to subsurface refugia may also have been important for survival. Drought extent is therefore likely to be a critical factor determining community persistence, as without sources of recolonists even the best‐adapted taxa could be vulnerable on supraseasonal timescales (Stubbington et al., 2016). Here, as with other experimental studies, the proximity of mesocosms to one another (20–80 cm), and thus the distance between drought‐affected habitats and recolonist sources, reflected the physical constraints of our site. This necessary simplification of metacommunity dynamics implies that our observations of drought impacts are, again, likely to be conservative (see Ledger, Harris, Armitage, & Milner, 2012 for a similar example). Nonetheless, in groundwater‐fed systems, where localized water abstraction effects and flow buffering by the aquifer can give rise to patchy drying patterns (Kendon et al., 2013; Westwood et al., 2006), dispersal between disturbed and undisturbed habitats could plausibly occur over short distances.

The taxa most adapted to drought are often small and *r*‐selected, as high reproductive rate and rapid maturation offer resilience to disturbance (Bonada, Dolédec, et al., 2007; Chessman, 2015; Ledger et al., 2012; Ledger, Edwards, Brown, Milner, & Woodward, 2011; Patrick & Yuan, 2017). However, body size also dictates drought resistance, as small size entails lower metabolic demand and facilitates easier access to suitable refugia (Griswold, Berzinis, Crisman, & Golladay, 2008; Ledger, Brown, Edwards, Milner, & Woodward, 2013; Woodward et al., 2016). Despite an abrupt increase in the prevalence of small body size as channels dried, high‐intensity drought did not favour all small taxa, and chironomids—which dominated TPG H—were particularly sensitive to drying. Certain chironomid subfamilies found in our study, such as Orthocladiinae, primarily comprise cold‐adapted stenotherms (Friberg et al., 2009; Worthington, Shaw, Daffern, & Langford, 2015) and wide temperature fluctuations would have constrained their presence in severely dewatered channels. The results reported here therefore accord with those of Nelson et al. (2017), who reported unexpected body size responses to stream warming attributable to variability in thermal preference, and suggest that *r*‐selection is a necessary but not sufficient condition for success during extreme drought.

Biotic adaptation to disturbance depends greatly on the predictability of the event (Lytle, Bogan, & Finn, 2008). Trait responses to regular seasonal drying in a historically intermittent stream may thus be expected to differ markedly from those expressed during an extreme drought in a perennial system. For example, we found no relationship between drought intensity and reproductive traits such as ovoviviparity and multivoltinism. These life history traits may be redundant when unpredictable drought nullifies adaptations to the historical disturbance regime, thus placing greater value—as observed here—on ad hoc behavioural responses and physiological resistance (de la Fuente et al., 2018; Lytle & Poff, 2004). Biotic responses to seasonal drying are often dominated by resilience mechanisms, which allow communities to recover following the predictable resumption of flow (Datry et al., 2014), but during prolonged droughts we might expect resistance strategies to become relatively more important for maintaining ecosystem functioning. Crucial mechanisms of community persistence in the face of future droughts, such as some of the Type T traits discussed here, might therefore fully reveal themselves only through an experimental approach subjecting species to true environmental extremes. Logistic and financial constraints meant we were unable to investigate community recovery from drought in the current study, so we could not formally test the relative importance of resistance vs. resilience strategies in the mesocosm communities here. However, the prevalence at high intensity of, for example, aerial respiration and dispersal suggests that both may be critical, a conjecture that can be addressed more rigorously in future work.

We suggest that our form of trait‐based approach, accounting for changes in both individual trait occurrence and functional group (TPG) abundance, could be used more widely to diagnose and predict functional responses to disturbance. The two analyses yielded distinct but complementary information: contrasting response patterns among individual traits provided direct, mechanistic insights into trait filtering under drought; while analysis of TPG abundance revealed early response thresholds that were not captured by the former method. These changes in TPG abundance could be considered analogous to the trait abundance shifts described by Boersma et al. (2016), whereby a decrease in the abundance, but not extirpation, of a particular trait combination (or here functional group) can provide an early warning signal of forthcoming functional extinctions (Säterberg, Sellman, & Ebenman, 2013). We therefore recommend that future traits‐based studies of drought look beyond community‐averaged response variables (e.g., individual trait occurrences), to ensure that potentially catastrophic functional impacts do not go undetected.

Ecological responses to extreme climatic events are typically highly idiosyncratic (van de Pol, Jenouvrier, Cornelissen, & Visser, 2017), so our ability to predict the ecological impacts of severe droughts will largely hinge on the mechanistic insights offered by controlled, manipulative experiments and traits‐based approaches. Understanding which traits confer resistance (and vulnerability) to extreme drought should allow for more targeted conservation efforts during water deficits. For instance, the tendency for most taxa with high physiological resistance to drying to be aerial dispersers underscores the importance of maintaining a network of refugia to act as sources of recolonists. More generally, the high sensitivity of many traits to drought intensification highlights their value as functional biomarkers for resistance and resilience at both species and community level, potentially supplementing existing taxonomy‐based biomonitoring metrics (e.g., DELHI index; Chadd et al., 2017).
