# Supplementary material for: Extreme drought pushes stream invertebrate communities over functional thresholds
Source: Glob Chang Biol. 2018 Nov 14;25(1):230–44. doi: 10.1111/gcb.14495 (PMC7379955; doi:10.1111/gcb.14495)
Supplement: Supplementary file 1 [file GCB-25-230-s001.docx]

**Extreme drought pushes stream invertebrate communities over functional thresholds**

**Supporting information**

**Table S1** Variance in the environmental data explained by the first two axes of the principal component analysis (PCA) used to formulate the drought intensity (DI) index, and the factor loadings of each environmental variable.

|  | PCA 1 | PCA 2 |
| --- | --- | --- |
|  | 93.8% | 3.2% |
|  |  |  |
| Flow | -0.98 | -0.02 |
| Wetted area | -0.97 | 0.21 |
| Water volume | -0.96 | 0.08 |
| Max temperature range | 0.96 | 0.26 |

**Table S2** List of references used to obtain length-mass regression equations for aquatic invertebrates.

| Benke, A. C., Huryn, A. D., Smock, L. A. & Wallace, J. B. (1999). Length-mass relationships for freshwater macroinvertebrates in North America with particular reference to the southeastern United States. *Journal of the North American Benthological Society*, **18**, 308–343. |
| --- |
| Burgherr, P. & Meyer, E. (1997). Regression analysis of linear body dimensions vs. dry mass in stream macroinvertebrates. *Archiv für Hydrobiologie*, **139**, 101–112. |
| Edwards, F. K., Lauridsen, R. B., Armand, L., Vincent, H. M. & Jones, J. I. (2009). The relationship between length, mass and preservation time for three species of freshwater leeches (Hirudinea). *Archiv für Hydrobiologie*, **173**, 321–327. |
| Mason, C. F. (1977). Populations and production of benthic animals in two contrasting shallow lakes in Norfolk. *Journal of Animal Ecology*, 46, 147–172. |
| Meyer, E. (1989). The relationship between body length parameters and dry mass in running water invertebrates. *Archiv für Hydrobiologie*, **117**, 191–203. |
| Smock, L. A. (1980). Relationships between body size and biomass of aquatic insects. *Freshwater Biology*, 10, 375–383. |

| Response variable | *F-*value | Deviance explained (%) | Threshold zone (DI range) |
| --- | --- | --- | --- |
| *Raw traits* |  |  |  |
| Small | 11.9*** | 67.4 | ≥0.66 |
| Medium | 3.85^ns^ | 37.3 | na |
| Large | 12.0*** | 68.2 | ≥0.74 |
| Vlarge | 0.18^ns^ | 0.94 | na |
| Multivoltine | 3.17^ns^ | 14.3 | na |
| Ovoviviparous | 3.25^ns^ | 14.6 | na |
| Resistant | 4.38* | 39.4 | ≥0.91 |
| Active aerial | 12.4*** | 58.8 | na |
| Active aquatic | 8.83** | 48.7 | na |
| Crawling | 12.4*** | 53.9 | na |
| Burrowing | 26.8*** | 73.1 | na |
| Tegument | 31.9*** | 79.7 | ≥0.71 |
| Spiracle | 10.5*** | 57.9 | ≥0.82 |
| Generalist | 14.9*** | 43.9 | na |
| Cold | 12.5*** | 68.6 | ≥0.64 |
| Eurythermic | 12.2*** | 67.4 | ≥0.64 |
| *TPGs* |  |  |  |
| A | 2.69^ns^ | 12.4 | na |
| B | 28.0*** | 81.9 | ≤0.39 |
| C | 0.72^ns^ | 10.8 | na |
| D | 21.2*** | 77.7 | ≥0.59 |
| E | 10.4*** | 57.8 | ≤0.22 |
| F | 52.0*** | 91.5 | ≥0.60 |
| G | 11.6*** | 66.2 | ≤0.33 |
| H | 2.53^ns^ | 41.4 | na |

**Table S3** Full GAM results for relationships between drought intensity and both raw traits and abundances of trait profile groups. ‘Deviance explained’ provides a measure of model performance, comparable to the R^2^ value in regression. ‘Threshold zone’ denotes the portion of the drought gradient where the slope of the fitted GAM is >1 or <-1. Significance value denotation is as follows: ns = non-significant (*P* > 0.05); *P* < 0.05^*^; *P* < 0.01^**^; *P* < 0.001^***^. All asterisked *F*-values are significant (*P* < 0.05) following the Benjamini & Hochberg (1995) procedure for controlling the false discovery rate.


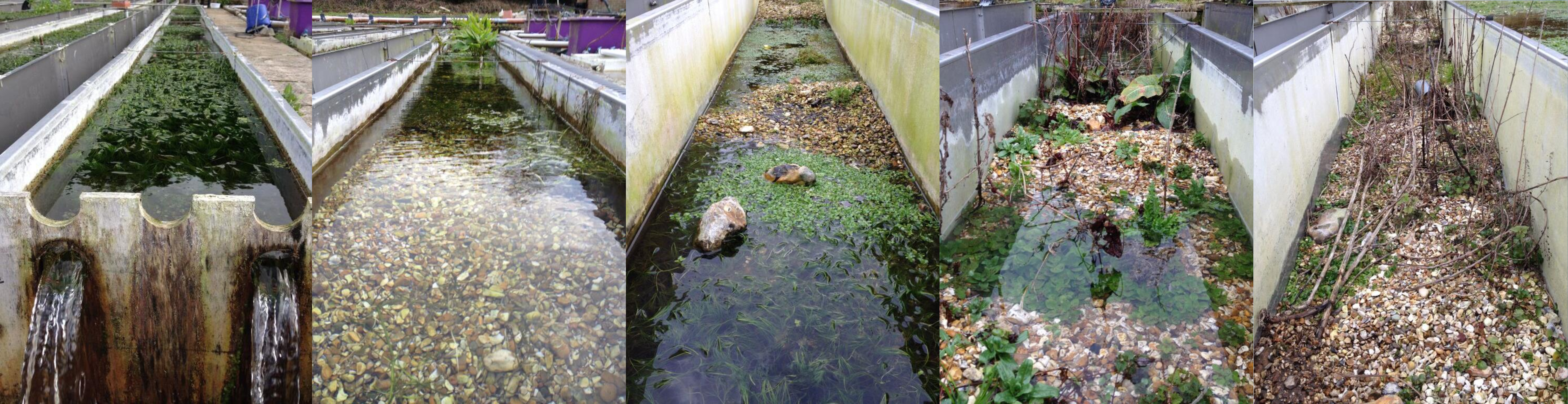


**Figure S1** Photos of a subset of the mesocosms showing the progressive change in conditions across the drought gradient, from flowing streams (first and second from left) to fragmented pools (middle and second from right) to dry streambeds (far right).


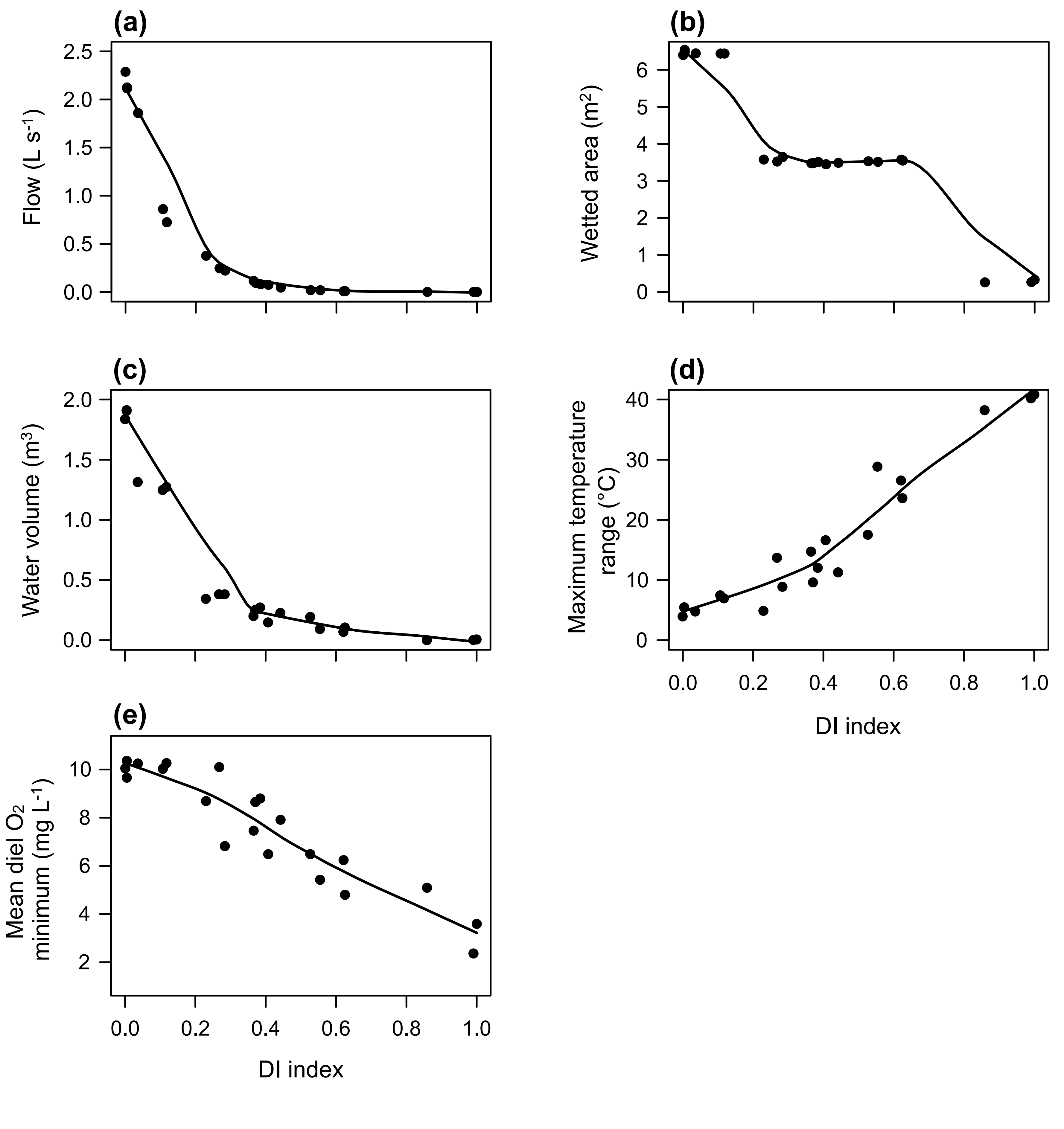


**Figure S2** Trends in abiotic variables along the drought gradient: (a) flow, (b) wetted area, (c) water volume, (d) maximum recorded temperature range and (e) mean daily minimum oxygen concentration. Fitted curves are lines of best fit given by LOESS smoothing (span=0.7). Two of the abrupt, critical habitat transitions described by Boulton (2003), namely pool fragmentation and loss of surface water, may be seen in (b).


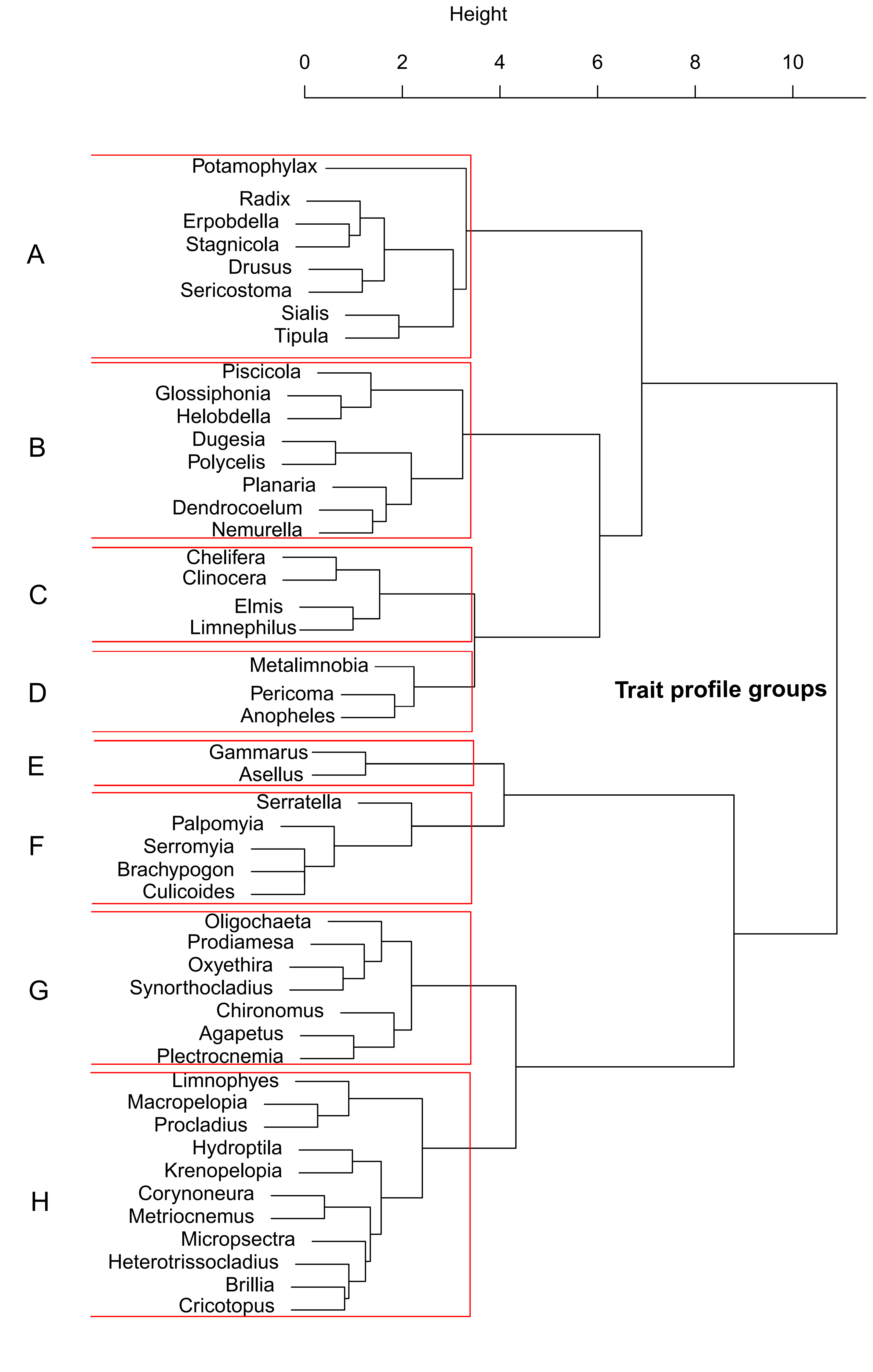
**Figure S3** Dendrogram showing the hierarchical clustering of taxa based on the traits listed in Table 2. Each red box delineates a trait profile group (TPG).


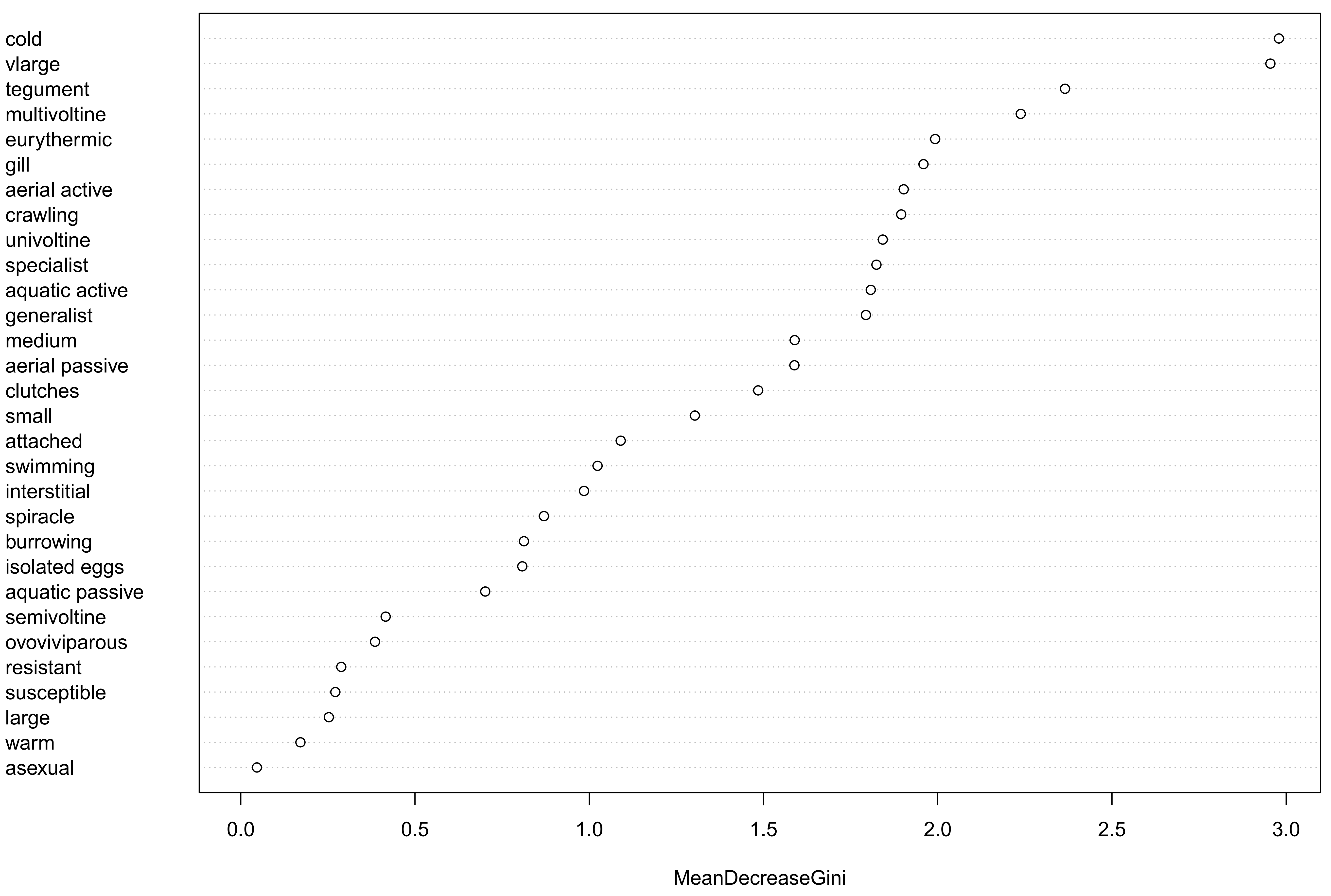


**Figure S4** Plot showing the overall (i.e. forest-wide: averaged across all decision nodes) importance of each trait in the random forest analysis. The higher the mean decrease in the Gini value, the more influential the trait for distinguishing between TPGs.
